# Lethal infective endocarditis due to *Streptococcus agalactiae* in a man with a history of alcohol abuse: A case report

**DOI:** 10.1097/MD.0000000000018270

**Published:** 2019-12-20

**Authors:** Myriam D’Angelo, Ilaria Boretti, Salvina Quattrocchi, Giovanni Alongi, Carmela Rifici, Francesco Corallo, Angela Magazù, Demetrio Milardi, Gaetano Cannavà, Placido Bramanti, Antonio Duca

**Affiliations:** a Istituto di Ricovero e Cura a Carattere Scientifico (IRCCS) Centro Neurolesi “Bonino Pulejo”, Messina; b Department of Biomedical, Dental Sciences and Morphological and Functional Images, University of Messina, Italy.

**Keywords:** alcohol abuse, neurological complications, *Streptococcus agalactiae*, vegetation in normal mitral valve

## Abstract

**Rational:**

Infective endocarditis (IE) is defined as an infection of the endocardial surface of the heart, which may include one or more heart valves, the mural endocardium.

**Patient concerns:**

A 53-years-old man with a history of alcohol abuse was admitted in hospital for fever, paroxysmal atrial fibrillation cardioverted by Amiodarone and pulmonary infection.

**Diagnosis:**

A case of recurrent severe endocarditis, with neurological complications both ischemic and hemorrhagic and heart failure caused by *Streptococcus agalactiae* in healthy man we reported.

**Interventions:**

Surgery was performed 2 weeks after admission.

**Outcomes:**

The onset of intracranial hemorrhage delayed second cardiac surgery and the patient died because of end-stage heart failure.

**Conclusions:**

Infective endocarditis caused by *S. agalactiae* is very rare, particularly in patients without underlying structural heart disease. This study showed that IE due to *S. Agalactiae* is a disease with high mortality when associated with neurological complication, heart failure but especially when it is recurrent and hits valve prosthesis.

## 1 Introduction

Despite improvements in medical and surgical therapies, infective endocarditis (IE) is still associated with poor prognosis and with considerable mortality and morbidity^[1–3]^ along with a high incidence of embolic events (EE), ranging from 13% to 50%.^[4–6]^ The main risk factors of IE are intracardiac device, intravenous drug use, degenerative valve disease and hemodialysis. Heart failure, stroke, multiorgan dysfunction syndrome and sepsis seem to be the most frequent situations leading to death. Rarely, IE affects patients without underlying structural heart disease.

Here, we present a case of severe IE caused by *Streptococcus agalactiae* in a healthy man without predisposing abnormalities but the alcohol abuse. *S agalactiae* is a Gram-positive coccus, β-hemolytic, of group B generally causing different diseases in newborns and pregnant women.^[7]^ It is also an important cause of invasive infection in older adults, particularly those with underlying chronic illness such as diabetes mellitus, malignancy and other causes of immunodeficiency.^[7–9]^

## 2 Case report

In November 2016, a 53-years-old man with a history of alcohol abuse was admitted in hospital for fever, paroxysmal atrial fibrillation cardioverted by Amiodarone and pulmonary infection. On admission laboratory findings included a high C-reactive Protein (CRP) concentration (25 mg/dl, normal value < 0.3 mg/dl) and abnormal white blood cell (WBC) count (18 × 10^9^/l). Grade II/III systolic murmur was audible at the apical area. Ceftriaxone and ciprofloxacin were started to treat suspected community-acquired pneumonia.

**Figure 1:**
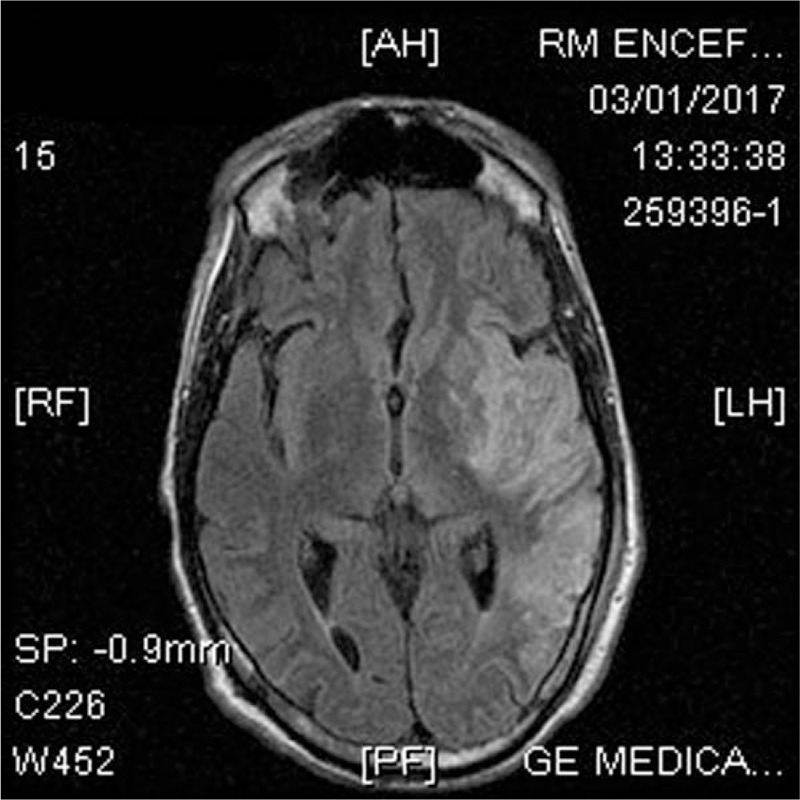
Brain MRI shows an acute infarction in the left hemisphere.

**Figure 2:**
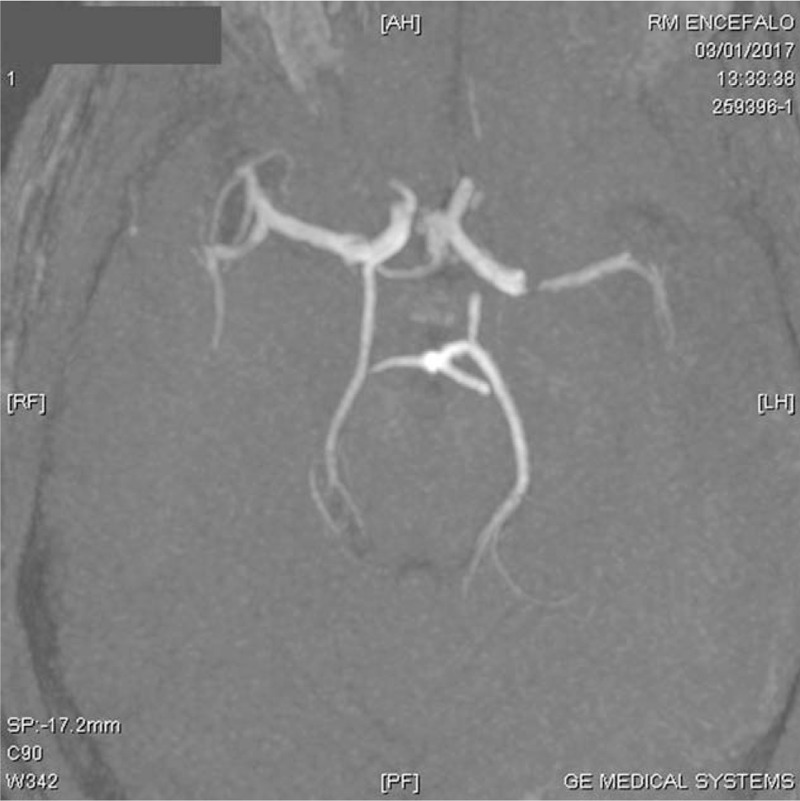
Angio MRI shows a filling defect of left middle cerebral artery.

Transthoracic echocardiography (TTE) revealed a large (25 × 15 mm), rounded, mobile, echo-dense mass attached to the ventricular side of a flail anterior mitral leaflet, due to ruptured primary chordae tendineae causing severe mitral regurgitation. Blood cultures revealed an infectious by *S agalactiae*. Piperacillin/tazobactam 4.5 gr 4 times a day was prescribed and 2 weeks later mitral valve replacement with a biological prosthesis CE n.25 was performed. The day after surgery, a physical examination showed global aphasia, dysphagia and right hemiparesis. Brain computed tomography (CT) showed a great ischemic area in the left hemisphere and brain magnetic resonance imaging (MRI) showed a large area of altered hyperintense signal in the long-TR sequences at the cortico-subcortical fronto-temporo-parieto-insular site (Fig. 1). Brain angio-MRI revealed a filling defect at the initial portion of left middle cerebral artery (Fig. 2).

Patient was treated with acetylsalicylic acid 300 mg and enoxaparin 6000 U.I. twice a day and discharged 1 month later with residual aphasia and right hemiparesis. The last transthoracic echocardiogram performed before discharge showed no vegetation on the mitral prosthesis and on the other valves.

In February, the patient was admitted to our neurological rehabilitation centre. On admission, he had a normal WBC count (6.8 × 10^9^/l), mild anemia (Hb 11 g/dl), normal CRP concentration and renal function. Stain for acid-fast bacilli and serologic screening for HIV, hepatitis B and hepatitis C were negative.

During the rehabilitation program, the patient presented with high-grade fever (38.5°C) and tachycardia. At physical examination bilateral diffuse coarse lung crepitations were found. Laboratory findings included high CRP concentration (20 mg/dl) and WBC count (16.4 × 10^9^/l). TTE showed a mobile, linear mass on the prosthesis and increased transprosthetic gradient.

**Figure 3:**
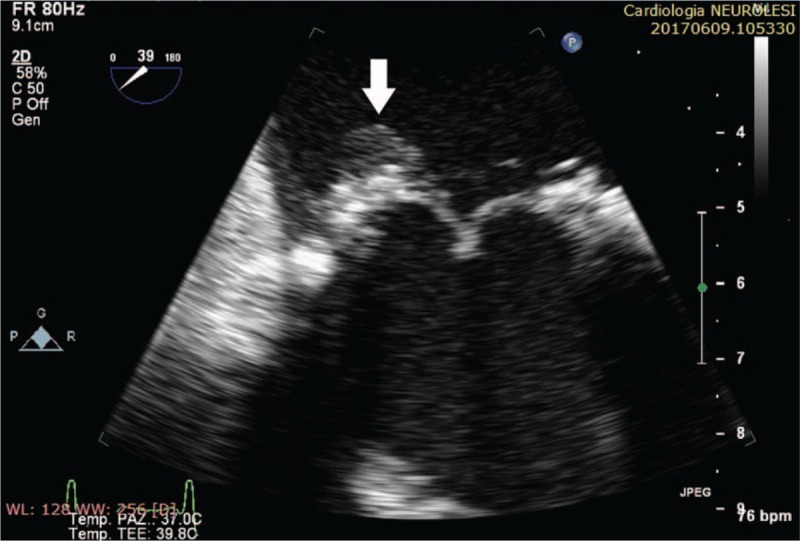
Transesophageal echocardiography shows a great vegetation on posteromedial portion of the mitral prosthetic ring. LA = left atrium.

**Figure 4:**
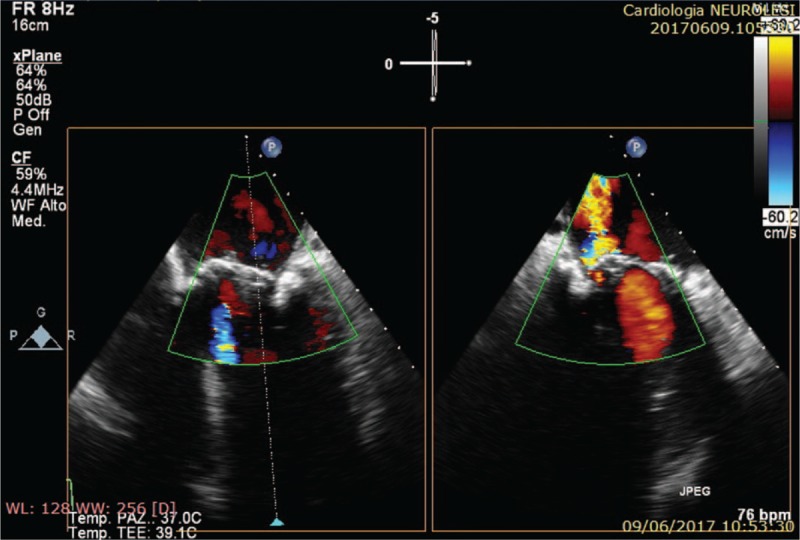
Transesophageal ecocardiography demonstrates a periprosthetic severe regurgitation.

TEE showed 2 echogenic vegetations, the greater attached to the posteromedial region of the mitral prosthetic ring, causing a periprosthetic severe regurgitation (Figs. 3 and 4).

Blood cultures showed a *S Agalactiae* infectious, imposing a triple combination antibiotic therapy with Daptomycin 10 mg/kg, Gentamicin 3 mg/kg once a day and Meropenem 1000 mg every 8 hours.

**Figure 5:**
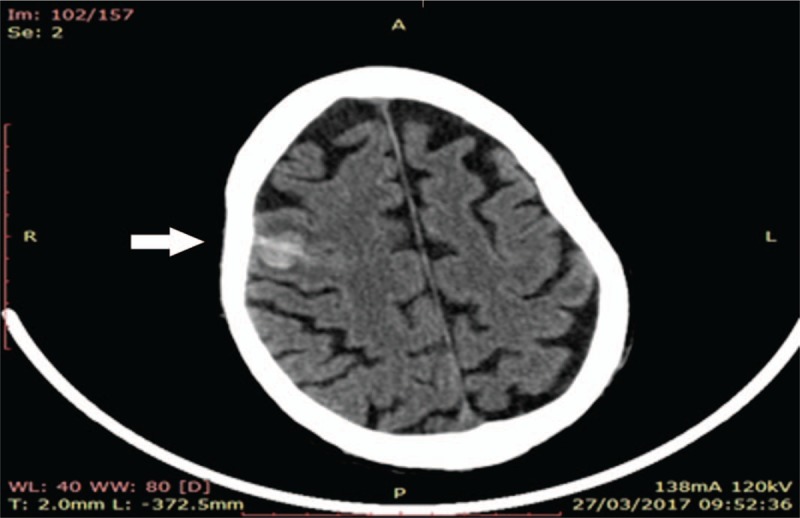
A brain CT reveals focal hemorrhage of 15 mm at the cortico-subcortical right frontal site.

The cardiac surgeon requested a new CT of the brain that revealed a focal hemorrhage of 15 mm at the cortico-subcortical right frontal site with hypodense halo (Fig. 5). Taking into account these new findings, in line with the current guidelines^[10]^ of the European Society of Cardiology, we decided to delay cardiac re-intervention and to continue antibiotic therapy and support treatment of heart failure.

One month later, hemoglobin dropped to 6.9 gr/dl with clinical deterioration and patient was transfused with red packed cells. Direct Coombs test was repeatedly positive and the patient received intravenous corticosteroids. Despite a reduction in the area of hyperdensity in the right frontal area, because of high surgical risk, no cardiac surgeon agreed to operate him. Clinical conditions were stable until June when fever reappeared again. Two months later the patient died because of end-stage heart failure.

## 3 Discussion

We presented a case of recurrent IE by *S agalactiae* with neurological complications in a patient with an history of alcohol abuse.

It is well known that embolism represents one of the most frequent and severe complications of IE, and it is accepted as one of major determinants of prognosis increasing morbidity and mortality. Up to 65% of EE involves the central nervous system.^[6,11]^ The risk of embolism seems to be particularly high during the first 2 weeks after diagnosis of IE as showed by Thuny et al^[12]^ which found that 71.4% of new-EE occurred during the first 15 days. These data have been confirmed by different other studies, such as the one of Vilacosta et al^[13]^ in a population of 217 patients with left-sided endocarditis showing that 65% of EE occurred during the first 2 weeks after initiation of antibiotic therapy and mainly involved the central nervous system.

The risk of embolization is likely to increase with vegetation sizes and mobility, and this is particularly true when the valve affected is the mitral and the pathogen is the *Staphylococcus aureus*.^[12–15]^

Vegetation length, age, renal failure, *S aureus* and moderate or severe cardiac heart failure (CHF) are considered to be predictors of 1 year mortality,^[12–18]^ while neurological complications are unfavorable outcome predictors as shown by Garcia-Cabrera et al^[19]^ in their retrospective study. In particular, they found that neurological complications determine an adverse prognosis, despite only cerebral hemorrhage and moderate-severe ischemic stroke are independent factors associated with mortality.

Another interesting prospective multicenter study involving 225 patients with IE showed that neurological failure at admission (defined by a Glasgow Coma Scale <10) was the main factor associated with death.

The appropriate timing of surgery for IE patients with cerebral complications remains controversial.^[20]^ Currently, it is recommended to delay valve surgery for ≥2 weeks in the case of severe ischemic strokes and ≥4 weeks for hemorrhagic events.^[10,21]^

The impact of valve surgery on the outcome in IE patients with cerebrovascular complications is still subject of debate. There are different opinions about the postoperative risks after an ischemic or hemorrhagic cerebrovascular event. An interesting study on 30 patients with intracranial hemorrhage investigated the risk related to timing valve surgery on postoperative neurological deterioration. The authors found that this risk was particularly low even in IE patients who underwent valve surgery within 2 weeks of intracranial hemorrhage onset.^[22]^

In a large published study,^[23]^ neurological deterioration decreased with time between the brain infarction and the valve replacement and there were reports of patients who had the valve replacement a few days after intracerebral hemorrhage with good outcome.

*S agalactiae* is frequently associated with morbidity and mortality among infants in the first weeks of life and with infection in pregnant women, however, it can be responsible of significant morbidity and mortality also among nonpregnant adults.^[7–9,24,25]^

Common presentations of *S agalactiae* in adults include skin and/or soft tissue infection, bacteremia without focus, pneumonia, osteomyelitis, meningitis, and endocarditis. Although the last 2 conditions are rare, they are associated with higher morbidity and mortality rate. In the last years, there was an increase in the incidence of Group B Streptococcal (GBS) infections.

There are different serotypes of *S agalactiae* and the pathogenesis of its infection is believed to be determined by its serotype. Based on the composition of the capsular polysaccharide, GBS can be divided into nine different serotypes. Serotypes Ia, Ib, II, and III have predominated in many parts of world^[26]^ but serotype V has emerged as an increasingly important pathogen.

In addition, different authors have reported a predominance of serotype V among nonpregnant adults with GBS infections.^[26–30]^

*S agalactiae* often causes infections in non-pregnant adult patients with chronic immunosuppressive disease, such as cancer, diabetes mellitus, HIV infection, and cirrhosis.

Our patient had only a history of alcohol abuse without additional predisposing factors for endocarditis or *S agalactiae* infection. Generally, alcoholic individuals are not necessarily more prone to endocarditis however a combination of immunodeficiencies with social and lifestyle situations increase their risk of endocarditis. Gram-positive bacteremia is uncommon but when present it is usually due to streptococci and *S aureus*.^[31,32]^

In 1972, Buchbinder and Roberts^[33]^ found that 14 (24%) out of 59 patients with fatal active infective endocarditis had chronic alcoholism. Frequently, the endocarditis was associated with pneumonitis or meningitis. In the post-mortem study of Snyder et al,^[34]^ the frequency of bacterial endocarditis was higher in cirrhotic patients than in non-cirrhotic patients.

The mortality rate of *S agalactiae* endocarditis is in the range of 20% when a combined medical and surgical strategy is performed and in the range of 40% to 50% with medical therapy alone.^[35,36]^

Herein, we show that IE due to *S Agalactiae* is a severe disease with high mortality when associated with neurological complication, heart failure but especially when it is recurrent and hits valve prosthesis.

## Author contributions

**Conceptualization:** Myriam D’Angelo, Ilaria Boretti, Antonio Duca.

**Investigation:** Demetrio Milardi.

**Methodology:** Salvina Quattrocchi.

**Resources:** Giovanni Alongi.

**Validation:** Gaetano Cannavà, Placido Bramanti.

**Visualization:** Carmela Rifici.

**Writing – original draft:** Angela Magazù.

**Writing – review & editing:** Francesco Corallo.

Antonio Duca orcid: 0000-0002-0353-7482.
